# Prospective evaluation of fluorescence-guided cystoscopy to detect bladder cancer in a high-risk population: results from the UroScreen-Study

**DOI:** 10.1186/2193-1801-3-24

**Published:** 2014-01-13

**Authors:** Marcus Horstmann, Severine Banek, Georgios Gakis, Tilman Todenhöfer, Stefan Aufderklamm, Joerg Hennenlotter, Arnulf Stenzl, Christian Schwentner, the UroScreen study group

**Affiliations:** Department of Urology, Friedrich Schiller University, Jena, Germany; Department of Urology, Eberhard Karls University, Tübingen, Germany

**Keywords:** Urothelial cancer of the bladder, Urine based tumor marker, Bladder cancer screening, NMP22, UroVysion, UroScreen, Cytology, Photodynamic diagnostics, Cystoscopy

## Abstract

**Objective:**

To prospectively evaluate the role of fluorescence-guided cystoscopy in a high-risk bladder cancer population undergoing screening based on a multi-marker panel of urine-tests (*UroScreen-study*).

**Patients and methods:**

UroScreen was conducted as a validation study for tumor markers within the frame of a health surveillance program of workers with occupational exposure to aromatic amines. Voluntary annual screens were done in 1,609 men. Cytology, quantitative NMP22® assay, and UroVysion (FISH) were applied to 7091 urine samples. Subjects with at least one positive urine-based tumor marker and/or persisting microscopic hematuria were offered fluorescence-guided (PDD) instead of white light cystoscopy. In case of suspicious findings histopathological evaluation by transurethral biopsy was performed. Data were statistically summarized and compared to tumors found by the standard algorithm of the screening study.

**Results:**

Twenty-two subjects with a mean age of 58 years (39–72) underwent PDD cystoscopy. Of those 3 had positive NMP22 tests, 14 positive FISH tests and 9 suspicious cytologies. Two had persisting microscopic hematuria only. PDD cystoscopy revealed enhanced unifocal fluorescence in 14. All had subsequent transurethral biopsy or resection. In total, 1 urothelial carcinoma (pTaG1, low grade) was diagnosed. In the other participants urothelial cancer of the bladder was ruled out. Chronic cystitis was revealed in 8 of 14 biopsies. No higher detection rate was found using PDD than with the standard algorithm of the *UroScreen* study in which 17 tumors were detected by white light cystoscopy.

**Conclusion:**

The use of PDD does not lead to a higher cancer detection rate in a high-risk screening population. Larger sample sizes may be needed to ultimately asses the value of PDD for bladder cancer screening.

## Introduction

White light cystoscopy is the current gold standard in the detection of bladder cancer. However, it is well known that also this method misses some papillary tumors (Grossman et al. 2007) and is limited in the detection of flat urothelial lesions representing carcinoma in situ (CIS) (Fradet et al. 2007). For such tumors the reported sensitivity of cystoscopy is low and ranges between 50 and 70% (vom Dorp et al. 2007). For an improved detection of CIS the additional use of urinary cytology is recommended because of its outstanding accuracy of approximately 95% for this particular condition (Rubben et al. 1979). Unfortunately, cytology cannot safely exclude low grade tumors and can be impaired by degenerative alterations, urinary tract infections and calculi. For further improvement of endoscopic tumor detection, fluorescence-guided cystoscopy or photodynamic diagnostics (PDD) has gained importance. Several studies investigated the efficacy of fluorescence-guided cystoscopy in the diagnosis of flat urothelial lesions and other tumors. Applying 5-aminolevulinic acid (5-ALA), Kriegmair et al. (1996) were able to demonstrate a significant increase of 50% in diagnosed CIS for the first time in 1996. In the meantime other groups confirmed these data with larger numbers of cases (Hungerhuber et al. 2007; Zaak et al. 2002). In two recent European and North American multi center studies a detection rate of 97% was recognized for CIS using hexaminolevulinic acid for PDD compared to only 58% using white light cystoscopy (Grossman et al. 2007; Fradet et al. 2007). In a North American study Grossman et al. (2007) achieved a 29% higher detection rate of Ta and a 15% higher detection rate of T1 tumors using PDD. Based on these data the use of PDD was included in the guidelines of the European Association of Urology (EAU) (Babjuk et al. 2013).

In parallel further progress in the development of urine based tumor makers has been made. Among them, NMP22 and UroVysion, have been approved for bladder cancer detection and surveillance by the FDA (Horstmann 2012). Both markers were recently evaluated together with cytology as screening tools in the largest prospective longitudinal cohort study to date (*UroScreen*) (Banek et al. 2012; Huber et al. 2012). This study was conducted between 2003 and 2010 and included 1609 male chemical workers with occupational exposure to carcinogenic aromatic amines. Because of their increased risk of developing bladder cancer, they undergo an annual occupational health care check including urine sediment and cytology. In addition to that, participants of the *UroScreen* study had an annual NMP22 and FISH Test. In case of gross hematuria, at least one positive urine based tumor marker or positive Cyt, subjects were recommended to undergo cystoscopy. This was done according to the study protocol in 202 cases using white light cystoscopy. In order to further optimize bladder cancer detection, however, a PDD cystoscopy was offered to all candidates requiring cystoscopy from Aug. 2009 to Aug. 2010. Herein, we report results and data from this sub-group that underwent fluorescence-guided cystoscopy.

## Material and methods

### Study subjects

The UroScreen has been previously described (Hungerhuber et al. 2007). In brief, between September 2003 and June 2010, 2,214 active and retired workers of two chemical companies who had been exposed to aromatic amines were offered an extended screening program with urine-based tumor markers (UroScreen-study) once a year in addition to the surveillance program of the German Social Accident Insurance. All participants signed a written informed consent, and IRB approval (No. 1/2003 V) was obtained before the conduct of this prospective study. Until the end of the study 1,772 participants took part in UroScreen at least once. In total, 7091 urine tests were collected throughout the entire program.

### Subgroup

Twenty two subjects, with either hematuria or a positive NMP22, FISH or Cyt test accepted the option of PDD instead of white light cystoscopy and took part in the present sub-study. All examinations were performed on a voluntary basis at the Department of Urology in Tubingen, Germany. All subjects were prepared for eventual transurethral resection in case of a tumor manifestation. Urethrocystoscopy was performed under local anesthesia one hour after hexaminolevulinic acid (Hexvix®, IPSEN) instillation. During cystoscopy the bladder was carefully inspected according to a standardized pattern first in white light and then in PDD mode. In case of small suspicious lesions biopsies were taken in the same setting (n = 11). If bigger lesions occurred, the subject was anesthetized and a transurethral resection performed (n = 3). Participants with a tissue resection received a urinary catheter and were hospitalized for 48 hours. All samples were evaluated histopathologically according to the 1973 and 2004 WHO guidelines (Bellmunt et al. 2009).

### Urine-based markers

For verification of the previous results all urine based tumor marker tests including cytology were repeated at the time of PDD cystoscopy. For the NMP22 test approximately 10 ml of urine were fixated immediately. These samples were stored at −20°C and conveyed (at 4–8°C) to a special facility within 48 hours for further workup. There, the fixated samples were centrifuged and NMP22 determined in the supernatant using the immunometric assay as provided by the manufacturer (NMP22®ELISA test, Matritech Inc., USA). Additionally equal parts of about 100 ml midstream urine were conserved for cytology and for FISH after fixation with Eposti (48% Methanol, 10% glacial acetic acid and 42% distilled water). For the FISH test samples were cyto-centrifuged, denaturized and hybridized according to protocol and the FISH multi-color probe mixture (centromere samples CEP 3, CEP 7 and CEP 17 as well as DANN-sample LSI 9p21) were used. Uncombined samples were removed during washing steps and the nuclei were counterstained using DAPI. All samples were anonymized. After arrival at the laboratory, they were stored at a temperature of 4–8°C and worked up within 24–48 hours. Microscopic evaluation was conducted in 25–30 morphologically abnormal cells from a urine sample. FISH results were positive if at least 4 cells showed an accrual of ≥2 chromosomes (3, 7 or 17) or if at least 12 cells did not exhibit a measurable signal for 9p21 (Inc. V. UroVysion Protocols 2000). Cyt was performed and evaluated as described and published before (Horstmann et al. 2009).

### Statistics

All clinical data and data of the urine based tests were summarized and compared using descriptive statistics (SPSS Version 20). Performance measures were calculated from the test in the last sample before diagnosis or the last screen if non-diseased. Multivariate generalized estimation equation models were applied to estimate odds ratios (OR) with 95% confidence intervals (CI) for factors influencing the tumor tests in repetitive samples from non-diseased men with information on urine status. Analyses were performed with SAS/STAT and SAS/IML, version 9.2 (SAS Institute Inc., Cary, NC, USA).

## Results

Twenty-two male subjects underwent diagnostic cystoscopy with PDD between August 2009 and August 2010. Their mean age was 58 years (range 39–72 years). Mean latency time between recommendation and performance of PDD cystoscopy was 2.7 (range 1.5–9) months. Of the 22 subjects, 3 had a positive NMP22 Test, 14 a positive FISH test and 9 positive Cyt at the time of last screening. Two participants had persistent microscopic hematuria alone. At the last screening, 5 of the subjects had an overlapping positive FISH and Cyt test, one an overlapping NMP22 and Cyt test and none an overlapping positive NMP22 and FISH tests. In none of the subjects all three markers were positive (Table 1).

**Table 1:** **Characteristics of the subjects who underwent diagnostic PDD in the UroScreen cohort**

	Age	N examination	Smoking status	Cytology	N positive UroVysion	N positive NMP22	N Micro-hematuria	N infection
1	65	3	Non smoker	0	0	1	0	1
2	61	5	Former smoker	G1	1	0	1	0
3	49	6	Smoker	0	1	0	0	0
4	66	6	Former smoker	0	0	4	1	1
5	67	6	Non smoker	0	2	0	0	0
6	49	5	Former smoker	G1	1	0	3	0
7	39	11	Non smoker	0	2	0	0	0
8	70	7	Non smoker	G1	0	0	0	0
9	52	6	Former smoker	0	1	0	0	0
10	51	5	Smoker	G1	2	0	1	1
11	42	6	Former smoker	0	1	0	0	0
12	66	6	Former smoker	G1	0	0	1	0
13	53	2	Smoker	G1	1	0	0	0
14	43	6	Non smoker	0	1	0	0	0
15	56	6	Non smoker	0	1	0	2	1
16	65	6	Non smoker	0	1	0	0	4
17	56	5	Former smoker	0	0	0	3	0
18	62	7	Former smoker	0	0	1	1	1
19	68	7	Former smoker	0	2	0	0	0
20	72	7	Former smoker	G1	1	0	1	2
21	65	7	Former smoker	0	0	0	2	3
22	68	7	Non smoker	G2	1	0	1	1

Fluorescence-guided cystoscopy revealed enhanced unifocal fluorescence in 14 cases and was completely normal in eight. Together with white light cystoscopy these lesions were considered to necessitate further histopathological evaluation. Therefore cold biopsies were taken from 11 and a TUR-B was performed in 3 subjects. The final histopathological evaluation revealed one patient with urothelial carcinoma (pTaG1, low grade) and 8 with chronic urocystitis (Table 1). The comparison of the maker results at the time of last screening with those obtained at the day of PDD cystoscopy, showed congruent results in 6 for NMP22, 5 for FISH, 7 for Cyt and 0 for microscopic hematuria. Smokers, former smokers and non-smokers did not show obvious correlations to results of the urine based tumor tests (Table 2). Five of the 8 patients histopathologically diagnosed with urocystitis had a positive FISH or Cyt test previously. Notably, the one patient with urothelial carcinoma had a positive FISH and Cyt test but initially a negative NMP22 test. In comparison to the rest of the UroScreen study in which 202 white light cystoscopies detected 17 tumors (8%), the detection rate of PDD cystoscopy, which detected one pTaG1 tumor in 22 patients, was not superior (4.5%). In the group of 202 patients with white light cystoscopy the time between urinalysis and diagnosis of BC varied from 0 to 26 months. Oft the 17 tumors in them four were low grade lesions, 13 high grade BC, and three papillomas. Age at diagnosis ranged in them between 38 and 78 years.

**Table 2:** **Results urine-tests and histopathological findings**

	Last screening before PDD-Cystoscopy			Time between last screening and pdd-Cystoscopy [months]	Screening during PDD-Cysoscopy			
	Cytology	NMP22	UroVysion		Cytology	NMP22	UroVysion	Histo-pathology
1	Negative	**14,12**	Negative	2	Negative	**75,97**	4	Urocystitis
			24.06.2009				21.08.2009	
2	**G1**	1,34	Negative	**4,5**	**G1**	Negative	**12**	Not done
			19.04.2010				**03.09.2009**	
3	Negative	1,14	**Positive**	2	Negative	**60,5**	**25**	Vasculitis
			22.06.2009				**25.08.2009**	
4	Negative	**13,73**	Negative	1,5	Negative	Negative	Negative	Urocystitis
			09.09.2009				23.10.2009	
5	Negative	5,77	**Postive**	2,5	Not done	Not done	Not Done	Urocystitis
			20.07.2009				01.10.2009	
6	**G1**	2,13	**Positive**	2	Negative	5,83	Negative	Urocystitis
			05.10.2009				08.12.2009	
7	Negative	2,33	**Positive**	3	Negative	**216,1**	**20**	Not Done
			24.08.2009				**24.11.2009**	
8	**G1**	1,35	Negative	1,5	Not done	Not done	Not done	Not Done
			07.10.2009				16.11.2009	
9	Negative	1,19	**Positive**	**2**	**G1**	Not done	Not done	Hyperplasia
			07.10.2009				15.12.2009	
10	**G1**	3,84	**Positive**	9	Negative	Not done	Not done	Not done
			17.03.2010				16.12.2009	
11	Negative	0,19	**Positive**	2,5	Negative	**34,59**	Negative	Urocystitis
			02.11.2009				18.01.2010	
12	**G1**	7,28	Negative	**1,5**	**G1**	**124,9**	**11**	Not done
			02.12.2009				**14.01.2010**	
13	**G1**	0,94	**Positive**	**2,5**	**G1**	1,57	Negative	Negative
			02.11.2009				13.01.2010	
14	Negative	5,31	**Positive**	4,5	Negative	**90,03**	Negative	Urocystitis
			09.09.2009				27.01.2010	
15	Negative	2,87	**Positive**	2	Negative	**58,35**	2	Negative
			14.12.2009				11.02.2010	
16	Negative	4,94	**Positive**	3	Negative	4,73	Not done	Urocystitis
			11.11.2009				11.02.2010	
17	Negative	4,46	Negative	1	Negative	5,7	Negative	Not Done
			18.01.2010				15.02.2010	
18	**G3**	**34,08**	Negative	2,5	Negative	Not done	0	Urocystitis
			03.03.2010				18.05.2010	
19	Negative	1,32	**Positive**	3,5	Not done	Not done	Not Done	Not Done
			01.03.2010				16.06.2010	
20	**G1**	2,6	**Positive**	**2**	**G1(G2)**	**22,36**	2	pTaG1
			17.05.2010				13.07.2010	
21	Negative	2,72	Negative	2,5	Negative	0,16	Negative	Not Done
			19.05.2010				08.08.2010	
22	**G2**	3,58	**Positive**	2	Negative	Not done	Not done	Negative
			12.08.2009				14.10.2009	

Data in Boldface represent positive tests.

## Discussion

The rationale for the present sub-study using PDD cystoscopy for tumor verification within the prospective *UroScreen-study* was to offer participants the most sensitive adjunct to cystoscopy. Hence, bladder evaluation secondary to positive urine based tumor marker evaluation should be optimized. Even though not yet used as reference standard in bladder cancer screening, the use of PDD has already proven to be superior to white light cystoscopy in standard tumor resection. In a meta-analysis published by Mowatt et al. (2010) in 2011, 31 studies (44 publications) of a total of 80 publications comparing white light resection to PDD were chosen for a more detailed analysis (27 on diagnostics, 4 on clinical efficacy). Altogether, in the 27 studies (2,949 patients) PDD showed a higher diagnostic sensitivity of 92% (95% KI: 80–100%) compared to white light resection with 71% (95% KI: 49–93). However, specificity was lower as compared to white light resection (57%; 95% KI: 36–79% vs. 72%; 95% KI: 47–96%). Because the increased tumor detection rates of PDD have a positive impact on the tumor-free rate as well as recurrence-free survival, as also shown in a recent review (Rink et al. 2013) and one meta-analysis (Burger et al. 2013), the current EAU guidelines on bladder cancer recommend PDD-guided TURB especially in suspected high grade bladder tumors and CIS (Babjuk et al. 2013). To further evaluate the significance of PDD it was offered in the present study from August 2009 to August 2010 instead of standard white light cystoscopy also in a screening situation. The hypothesis was that with the use of PDD some of the “false positive” marker tests would indeed become positive improving their predictive capacity. Hence, a higher detection rate of urothelial cancer in a screening population was suspected. However, in the present study the cancer detection rate remained low with only one pTaG1 tumor and no CIS detected. According to our interpretation this finding was most likely related to the unexpectedly low incidence of urothelial cancer in the whole *UroScreen* study (Banek et al. 2012; Huber et al. 2012). There are several potential reasons for such a low incidence rate: relatively young age of the screened population, cumulative exposure to carcinogenic amines was lower than assumed. Whereas in the *UroScreen* study the mean age was 62 years, mean age of bladder cancer patients generally ranges from 65–75 years (Pirastu et al. 1996; Otto et al. 2010) and only 5% of patients with bladder cancer are younger than 45 years (Pelucchi et al. 2006).

However, even though the present PDD sub-study did not prove an increased tumor detection rate and the number of its participants is too small for final statements, it nevertheless reveals some important aspects of bladder cancer screening based on urinary tumor markers. Firstly, it is quite obvious that the evaluated marker sensitivity not only depends on its true performance but also on the performance of the verification tool. Even though not shown in the present study one excellent example for this is given by CIS. In this condition, as stated above, the current sensitivity of white light cystoscopy is considered to be at about only 50–60% and in PDD cystoscopy at about 90% (Zaak et al. 2002). Using the same tumor marker in the same subjects but evaluating it with either white light or PDD cystoscopy would result in extremely different sensitivities and specificities. As this assumption, however, could not be verified in the present study further comparative evaluation regarding that aspect are required. Secondly, the current study shows that bladder cancer screening in otherwise healthy subjects remains hampered by the limited reliability of the available tumor marker tests. According to the present data this is clearly demonstrated by the relatively small numbers of overlapping positive and negative test results at two different points of time. Whereas in all 22 participants at least one marker including microscopic hematuria was positive, urine tests were completely normal in 6 participants at the time of re-testing. This high variability of marker tests results in conflicting data leading to an unnecessary diagnostic workup to rule out suspected cancer. Hence, additional PDD cystoscopy bears the risk of creating further false positive results. In the present study enhanced unifocal fluorescence was observed in 14/22 participants. However, only one subject had urothelial cancer! Even if this was most probably related to the primary aim of the present study, explicitly not to miss a tumor, this negative aspect of an increase of false positive cystoscopies using PDD diagnostic has to be taken very seriously.

Bladder cancer represents a highly heterogenous group of tumors growing and progressing at different speeds. Therfore, the optimal frequency of re-testing over time has to be carefully chosen in order not to miss too many tumors during screening intervals and not to exaggerate the screening intensity at the same time.

Interestingly in this context, urocystitis was frequently observed in the present patient population. However, the extent to which chronic urocystitis can evolve to dysplasia and finally urothelial carcinoma is unclear. Recurrent infections can induce the development of bladder cancer, in particular squamous cell carcinoma. Especially patients with long-term urinary catheters or cystolithiasis are affected (Wynder and Goldsmith 1977). In a retrospective study by Otto et al. (2010), 7% of patients with initial urocystitis developed bladder cancer during the follow-up period. Data of the present study may support this hypothesis as there was a strong association between urocystitis and a positive UroVysion test anticipating tumor manifestation (Laudadio et al. 2005; Gofrit et al. 2008). Additionally, the fact that no relevant confounding factors could be established regarding the UroVysion test in the UroScreen study support this hypothesis (Banek et al. 2012). Regarding the high number of positive NMP22 test results during PDD examination they can most likely be explained by the mechanic manipulation in the urinary tract during cystoscopy or the intravesical instillation of Hexvix^R^ prior to the intervention. This can result in the release of NMP22 due to an increased cell turnover (Poulakis et al. 2001; Konety 2006). For this reason these test results have to be interpreted with caution. Regarding Cyt, the high divergence between the two time points of examinations seems to be mainly attributed to the interobserver differences.

In summary, PDD was not able to pick up a higher number of bladder cancer cases in the *UroScreen-study* population. Since PDD cystoscopy still represents the most sensitive evaluation tool for the presence of bladder cancer further studies related to positive urinary tumor marker tests are warranted.
